# Effects of mechanical in-exsufflation in preventing postextubation acute respiratory failure in intensive care acquired weakness patients: a randomized controlled trial

**DOI:** 10.5935/2965-2774.20230410-en

**Published:** 2023

**Authors:** Philippe Wibart, Thomas Réginault, Margarita Garcia-Fontan, Bérangère Barbrel, Clement Bader, Antoine Benard, Verônica Franco Parreira, Daniel Gonzalez-Antón, Nam H. Bui, Didier Gruson, Gilles Hilbert, Roberto Martinez-Alejos, Frédéric Vargas

**Affiliations:** 1 Department of Medical Critical Care, Hôpital Pellegrin, Centre Hospitalier Universitaire de Bordeaux, Université de Bordeaux - Bordeaux, France; 2 Biostatistics Department, Bordeaux University Hospital - Bordeaux, France; 3 Department of Physiotherapy, Universidade Federal de Minas - Belo Horizonte (MG), Brazil; 4 Saint Eloi Department of Critical Care Medecine and Anesthesiology, Montpellier University Hospital, - Montpellier, France

**Keywords:** Weaning, Ventilator weaning, Respiratory insufficiency, Insufflation, Muscle weakness, Intensive care units

## Abstract

**Objective:**

We hypothesized that the use of mechanical insufflation-exsufflation can reduce the incidence of acute respiratory failure within the 48-hour post-extubation period in intensive care unit-acquired weakness patients.

**Methods:**

This was a prospective randomized controlled open-label trial. Patients diagnosed with intensive care unit-acquired weakness were consecutively enrolled based on a Medical Research Council score ≤ 48/60. The patients randomly received two daily sessions; in the control group, conventional chest physiotherapy was performed, while in the intervention group, chest physiotherapy was associated with mechanical insufflation-exsufflation. The incidence of acute respiratory failure within 48 hours of extubation was evaluated. Similarly, the reintubation rate, intensive care unit length of stay, mortality at 28 days, and survival probability at 90 days were assessed. The study was stopped after futility results in the interim analysis.

**Results:**

We included 122 consecutive patients (n = 61 per group). There was no significant difference in the incidence of acute respiratory failure between treatments (11.5% control group *versus* 16.4%, intervention group; p = 0.60), the need for reintubation (3.6% *versus* 10.7%; p = 0.27), mean length of stay (3 *versus* 4 days; p = 0.33), mortality at Day 28 (9.8% *versus* 15.0%; p = 0.42), or survival probability at Day 90 (21.3% *versus* 28.3%; p = 0.41).

**Conclusion:**

Mechanical insufflation-exsufflation combined with chest physiotherapy seems to have no impact in preventing postextubation acute respiratory failure in intensive care unit-acquired weakness patients. Similarly, mortality and survival probability were similar in both groups. Nevertheless, given the early termination of the trial, further clinical investigation is strongly recommended.

**Clinical Trials Register:**

NCT 01931228

## INTRODUCTION

Intensive care unit-acquired weakness (ICUAW) is a critical condition presented in ICU patients and characterized by generalized muscular weakness, including weakness in the peripheral muscles from the upper and lower limbs.^(1)^ According to De Jonghe et al.,^(2)^ 25% of patients admitted to the ICU who remain under mechanical ventilation for more than seven days present with ICUAW. Respiratory muscles can also be affected; indeed, limb weakness is correlated with an impairment of respiratory muscles and negatively impacts vital capacity and maximum inspiratory pressure.^(3)^ Thus, the presence of ICUAW is independently associated with an increased weaning period, delayed extubation and inefficient cough.^(4)^

The inefficient cough in ICUAW patients is due to the presence of an endotracheal tube and weakness in abdominal muscles, hindering their ability to mobilize pulmonary secretions, promoting airway mucus retention and increasing the risk of respiratory failure.^(5)^ Of note, in clinical practice, peak cough expiratory flow (PCEF) > 270L/minute is considered an indicator of an effective cough.^(6-8)^

Post-extubation acute respiratory failure (ARF) is a perilous situation characterized by dyspnea and hypoxemia, which can require reintubation in the worst-case scenarios. Inefficient airway clearance is one of the most critical factors leading to this failure.^(9)^ Moreover, ARF after extubation is a risk factor for increased nosocomial pneumonia, ICU length of stay, and mortality.^(10)^ Strategies to manage airway clearance to prevent postextubation ARF in these patients are crucial. However, it is estimated that 30% of patients develop ARF within 48 hours post-extubation.^(10)^

Current management of airway clearance in the ICU includes conventional chest physiotherapy (CCPT) based on manual compressions of the rib cage and abdominal wall to modulate expiratory flows and manually assisted cough.^(11,12)^ Furthermore, over the last 20 years, mechanical insufflation-exsufflation (MI-E) devices have been embraced as a novel adjuvant tool to CCPT to improve airway clearance.^(12-15)^ Mechanical insufflation-exsufflation provides mechanical insufflation with a positive pressure followed by a rapid shift to a negative exsufflation pressure, increasing the PCEF and simulating cough.^(16)^ Studies assessing patients with neuromuscular diseases have demonstrated that MI-E can generate a higher PCEF than spontaneous cough,^(6,12)^ be more effective than airway suctioning,^(17)^ prevent episodes of respiratory failure,^(16)^ and decrease the number of hospitalizations^(18)^ and risk of death.^(19,20)^ Owever, studies evaluating patients in the ICU are scarce. Gonçalves et al.^(21)^ assessed the efficacy of MI-E performed during a spontaneous breathing trial and immediately after extubation to prevent reintubation in patients developing ARF after extubation. They found a decrease in the reintubation rates and length of ICU stay when MI-E was associated with a standard extubation protocol.

Thus, we hypothesized that the use of MI-E can reduce the incidence of ARF within the 48-hour postextubation period in ICUAW patients.

## METHODS

### Study design

A randomized, controlled, monocentric open-label, parallel clinical study was conducted in a 25-bed medical ICU unit in France. All consecutive patients meeting the inclusion criteria were enrolled and randomized into a Control Group (performing CCPT) or MI-E Group (CCPT associated with MI-E). Randomization was centralized via secured electronic software (SAS v 9.3, SAS Institute Inc, Cary, NC, USA) balanced in a 1:1 ratio using a size block of 6 or 8 randomly and was performed one hour after extubation to guarantee that patients did not present immediate ARF due to upper airway obstruction.

The presented study was approved by the corresponding Institutional Review Board (no 2011-A01459-32), which waived the need for informed consent.

### Population

All patients diagnosed with ICUAW between May 2012 and January 2015 were considered eligible for this study. The inclusion criteria were critically ill adult patients (age ≥ 18 years), under invasive mechanical ventilation > 48 hours, presenting a Glasgow coma scale score of > 10 and an ICUAW with Medical Research Council (MRC) score ≤ 48 over 60 points the day of extubation prior to removing the artificial airway (Supplementary material).^(2)^ Similarly, the exclusion criteria were respiratory and/or hemodynamic instability; contraindication for the use of a facial mask (i.e., dysmorphia, facial skin lesions); recent upper gastrointestinal surgery or bleeding; severe ventricular arrhythmia; uncontrollable vomiting; upper airway obstruction (i.e., laryngeal edema); tracheomalacia; severe sepsis; undrained pneumothorax; tracheotomized patients; and history of bullous emphysema.

### Interventions

In both groups, patients received two sessions daily for the first 48 hours performed by a group of trained ICU physiotherapists, with a minimum interval of 4.5 hours between sessions. Additional CCPT could be performed if the patient presented a decrease in transcutaneous oxygen saturation (SpO_2_) of 5% with respect to the baseline value; abnormal adventitious breath sounds (i.e., crackles) or abolition of normal breath sounds; and/or worsening of arterial blood gases or abnormal chest X-rays.

Similarly, in the immediate postextubation period, we implemented all necessary strategies to ensure successful extubation and to avoid short-term respiratory complications, including noninvasive ventilation (NIV) (Supplementary material), aerosol and oxygen therapy.

### Control Group

Patients in the Control Group received standard treatment with CCPT. All the sessions were performed with patients in the semirecumbent position between 30 - 45o above horizontal. Manual compressions were based on a combination of three techniques: first, a slow expiratory technique to allow distal secretions to promote proximal airways;^(11,22,23)^ a forced expiratory technique to displace proximal secretions to the upper airway^(24)^ and manually assisted cough to facilitate expectoration.^(6,25,26)^ These techniques were executed and repeated until changes in breath sounds were perceived. Moreover, tracheal suctioning was performed if necessary.

### MI-E Group

In this group, patients received CCPT as aforementioned associated with a CoughAssist® MI-E device (Respironics INC, Murraysville, PA, USA) through a facial mask (AcuCare™F1-0, ResMed, Bella Vista, AUS) and a disposable filter.

For the first session, MI-E was set in manual mode, and insufflation-exsufflation pressures were set at +20/-20cmH_2_O to allow patients to familiarize themselves with the device and were gradually increased over time to +40/-40cmH_2_O at the end of the first-day intervention. However, if lung compliance or the effectiveness of cough was greatly diminished, negative exsufflation pressures up to -60cmH_2_O were tolerated to provide an efficient cough. At the end of that first session, the automatic mode was set to allow physiotherapists to perform CCPT during MI-E. In this mode, the insufflation-exsufflation time was set at 3 seconds each and a 1-second pause between cycles.

Each session included three to five series of five insufflation-exsufflation cycles. For the Control Group, tracheal suctioning was performed if necessary.

Beyond 48 hours of the primary outcome, patients benefitted from a single session per day - according to the randomization group. Similarly, additional sessions were performed if the patient met at least one of the aforementioned criteria. This procedure was repeated until patients presented an MRC score > 48^(2)^ or a PCEF ≥ 270L/minute;^(8)^ or patients were discharged from the ICU, or until a follow-up of 28 days in the ICU, whatever was first.

### Measurements

The primary outcome was the incidence of ARF within 48 hours after extubation. ARF is defined as the presence of at least two of the following criteria: respiratory rate > 35 or < 12bpm; clinical signs of respiratory distress (i.e., cyanosis, sweating, increased use of accessory respiratory muscles or paradoxical breathing); hypercapnia associated with respiratory acidosis (PaCO_2_ ≥ 10% of preextubation PaCO_2_ value and pH < 7.35) and oxygen therapy (≤ 3L/minute); and hypoxemia (SpO_2_ < 90%, PaO_2_ ≤ 60mmHg, or PaO_2_/FiO_2_ ≤ 120) under oxygen therapy ≥ 6L/minute or FiO_2_ ≥ 50% with a Venturi mask.

The secondary exploratory outcomes assessed were reintubation rate 48 hours after extubation and were based on one major criteria (cardiorespiratory arrest, respiratory pauses with changes in state of consciousness, hemodynamic instability - systolic blood pressure lower than 70mmHg -, cardiac arrhythmia poorly tolerated) or two minor criteria (ventilation inefficiency due to agitation or major leakage under NIV, respiratory rate > 35 breaths/min, pH < 7.25 or PaO_2_/FiO_2_ ≤ 120 despite using NIV and presence of another organ failure). Similarly, the postextubation ICU length of stay and mortality were measured during the ICU stay or until Day 28, whatever arrived first, and the probability of survival until Day 90 was also assessed.

Finally, we recorded the evolution during the 4 preprogrammed sessions (Day 1 and Day 2) of MRC and PCEF; the number of patients needing at least one airway suctioning during the sessions, the number of patients needing at least one additional session of physiotherapy and patient comfort. The baseline MRC score was obtained as previously described, and the baseline PCEF was obtained immediately after extubation and prior to the first intervention (i.e., Day 1). Similarly, these parameters were assessed before the first session of each day (i.e., Day 2) prior to each intervention. Medical Research Council was assessed as described and validated previously,^(2,27)^ and the PCEF was measured via a mouthpiece connected to a peak flow meter (Mini-Wright, Clement Clarke Int., Harlow, UK). Patients were required to perform maximal inspiration until reaching total lung capacity, followed by a cough. After three reproducible measures and without leaks or incoordination, the best value was registered.^(6,28)^ Patient comfort was evaluated after each session with a comfort visual analog scale.^(29)^

### Statistical analysis

We report the mean (standard deviation) or the median (interquartile range) for continuous variables, while categorical variables are presented as the number and percentage of patients. Sample size calculation was based on the expected difference in the ARF rate between the MI-E and control groups 48 hours after extubation. We considered the initial risk of ARF 48 hours after extubation in the control group to be 30% (a usual finding in our ICU). This reflected our hypothesis of a 50% relative decrease in ARF in the intervention group, as we informed the statistician that we expected a very high impact of MI-E as an adjunctive treatment, according to our clinical experience. With a 5% bilateral α risk, we needed to enroll 240 participants (120 per group) to achieve 80% power in an intent-to-treat analysis. Missing data for the primary outcome were considered failures. An independent scientific committee validated each exclusion.

Nominal variables were compared by using the Chi-square test or Fisher’s exact test, as appropriate. Continuous variables were compared by using Student’s t test or the Wilcoxon signed rank test, when appropriate. Adjusted analyses were performed considering available data and using logistic regression models.

Survival analyses were performed using the log-rank test and expressed by Kaplan‒Meier curves. Semiparametric Cox regression models were used to perform adjusted analyses. Statistical analyses were implemented in SAS® Software (SAS v9.3, SAS Institute Inc., Cary, NC, USA). For all performed comparisons, a p value of ≤ 0.05 was considered statistically significant.

## RESULTS

**Figure 1:**
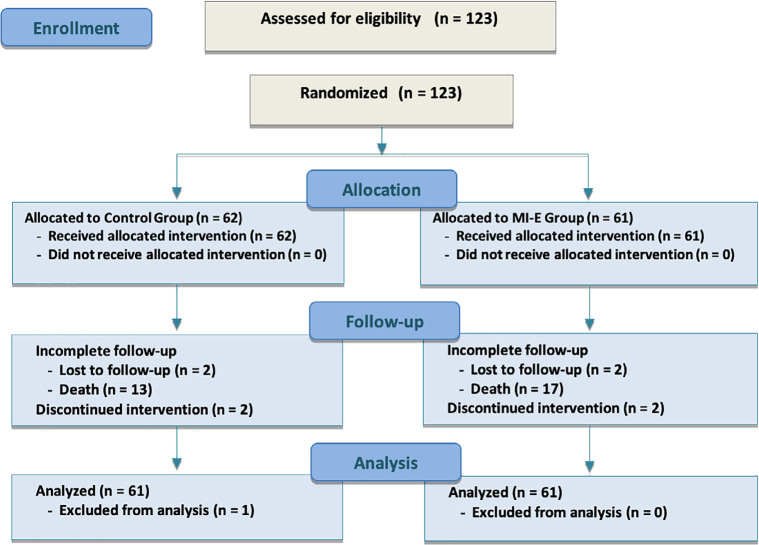
Study flow chart.

A total of 123 consecutive patients who had been under invasive mechanical ventilation for at least 48 hours before extubation were randomized for the study. One included participant presented a distorted MRC measurement at inclusion due to sedation. Consequently, he did not reach a major eligibility criterion and was excluded from the analysis. Thus, analysis for the primary outcome was performed with data from 122 patients, and in the secondary analyses, a maximum of 121 patients was considered (Figure 1). Reasons for intubation were sepsis or septic shock (n = 48; 39.7%), pneumonia (n = 39; 32.2%), respiratory complications related to a neurological/neuromuscular disorder (n = 13; 10.7%), acute respiratory distress syndrome (n =10; 8.3%), acute respiratory failure (n = 7; 5.8%) and cardiac failure (n = 4; 3,3%) without any significant differences between the groups.

MI-E Group - conventional chest physiotherapy + mechanical insufflation-exsufflation.

Recruitment was stopped in January 2015 after an effectiveness interim analysis at 50% of inclusions by a Data and Safety Monitoring (DSMB) committee. The DSMB reviewed the results of 123 patients and recommended stopping the study due to the low incidence of ARF in both groups and the lack of benefits in any major secondary outcome. Furthermore, they concluded that even if the sample size was attained, the probability of finding a significant difference in the primary outcome would be only 6.64%.

After extubation, a total of 463 sessions were performed: 231 in the control group and 232 in the MI-E Group, and only 54 patients (23.4%) in each group received the 4 preprogrammed sessions. Only one patient presented a desaturation during a session in the Control Group. In the MI-E Group, the majority of the sessions were performed with an insufflation pressure set at +40cmH_2_O (n = 147; 63.4%) and an exsufflation pressure of -40cmH_2_O (n = 165; 71,1%). At least three series of 5 cycles were performed during 64% of the MI-E sessions (Table 1).

**Table 1:** Demographic and clinical characteristics of patients with intensive care unit-acquired weakness at baseline

Demographic and clinical data	Control Group (n = 61)	MI-E Group (n = 61)^*^
Age (years)	65 (58 - 75)	67 (59 - 77)
Males	40 (65.6)	36 (60.0)
SAPS II	59 (46 - 73)	69 (59 - 82)
MRC score	33 (25 - 40)	36 (24 - 42)
Corticosteroid therapy	31 (52.5)	32 (55.2)
Curare therapy	13 (22.4)	18 (31.0)
Causes for ICU admission		
Pulmonary disease	23	25
Sepsis	16	20
Coma	14	8
Heart failure	2	4
Acute/chronic respiratory failure	3	0
Others causes	3	4
Blood gas		
pH	7.46 (7.42 - 7.49)	7.46 (7.43 - 7.50)
PaO_2_ (Kpa)	11.0 (9.0 - 11.9)	11.2 (9.7 - 13.8)
PaCO_2_, (Kpa)	4.7 (4.1 - 5.4)	4.8 (4.0 - 5.3)
HCO_3_- (mmol/L)	24.2 (22.0 - 27.7)	23.6 (20.5 - 28.2)
SaO_2_ (%)	97 (96 - 98)	98 (97 - 99)
Pao_2_/ Fio_2_	275 (225 - 321)	302 (230 - 368)
Patients under NIV	53 (86.9)	44 (72.1)
Patients with high mucus quantity	23 (37.7)	21 (34.4)
Mean blood pressure (mmHg)	91 (82 - 101)	92 (81 - 101)
Heart rate (bpm)	89 (78 - 105)	91 (78 - 101)
Respiratory rate (bpm)	23 (20 - 27)	23 (18 - 26)
SpO_2_ %	97 (96 - 99)	98 (94 - 99)
VAS comfort	7 (5 - 8)	6 (5 - 8)

MI-E Group - conventional chest physiotherapy + mechanical insufflation-exsufflation; SAPS II - Simplified Acute Physiological Score II; MRC - Medical Research Council; ICU - intensive care unit; PaO_2_ - partial pressure of oxygen; PaCO_2_ - partial pressure of carbon dioxide; HCO_3_ - bicarbonate; SaO_2_ - oxygen saturation; FiO_2_ - fraction of inspired oxygen ; NIV - noninvasive ventilation; SpO_2_ - transcutaneous oxygen saturation; VAS - visual analog scale.

* Data completely missing for one patient after 61 patients were included in this group. Results expressed as median (interquartile range 25% - 75%), n (%) or n.

### Primary outcome

The incidence of ARF within 48 hours after extubation in the MI-E group appeared to be higher than that in the control group, although this result was not significant (16.4% *versus* 11.5%, respectively; p = 0.60). The secondary analysis after adjustment for the baseline Simplified Acute Physiological Score II (SAPS II) score (Table 1) did not show a higher incidence of ARF (OR = 0.86, 95%CI = [0.17 - 4.40]).

### Secondary outcomes

**Figure 2:**
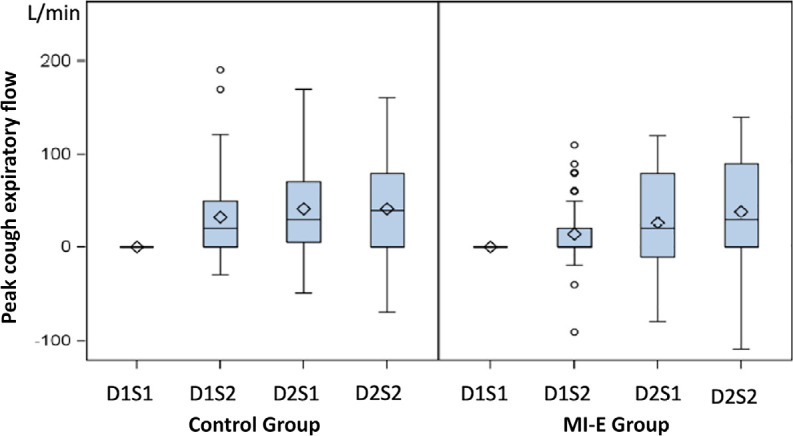
Peak cough expiratory flow evolution.

The need for reintubation 48 hours postextubation was similar between the groups (p = 0.27). Similarly, the need for at least one airway suctioning during treatment sessions was significantly lower in the MI-E Group than in the Control Group (p = 0.01) (Table 2) but no signification evolution in PCEF was observed (Figure 2).

**Table 2:** Primary and secondary outcome data

Outcomes	Control Group (n = 61)	MI-E Group (n = 61)^*^	p value
Primary outcome			
Acute respiratory failure on 48 hours after extubation	7 (11.5)	10 (16.4)	0.602
Secondary outcomes			
Reintubation on 48 hours after extubation	2 (3.6)	6 (10.7)	0.271
Postextubation ICU length of stay (days)	3 (2 - 7)	4 (2 - 7)	0.329
Patients needing at least one airway suctioning during session	19 (31.1)	7 (11.7)	0.010†
Patients needing at least one additional session	9 (14.8)	7 (11.7)	0.789
VAS comfort	5.5 (5.0 - 7.0)	5.9 (5.0 - 7.5)	0.641

MI-E Group - conventional chest physiotherapy + mechanical insufflation-exsufflation; ICU - intensive care unit; VAS - visual analog scale.

* Data was completely missing for one patient after 61 were included in this group for analyzing the secondary outcomes; † p < 0.05. Results expressed as n (%) or median (interquartile range 25% - 75%).

MI-E Group - conventional chest physiotherapy + mechanical insufflation-exsufflation; D - day; S - session.

**Figure 3:**
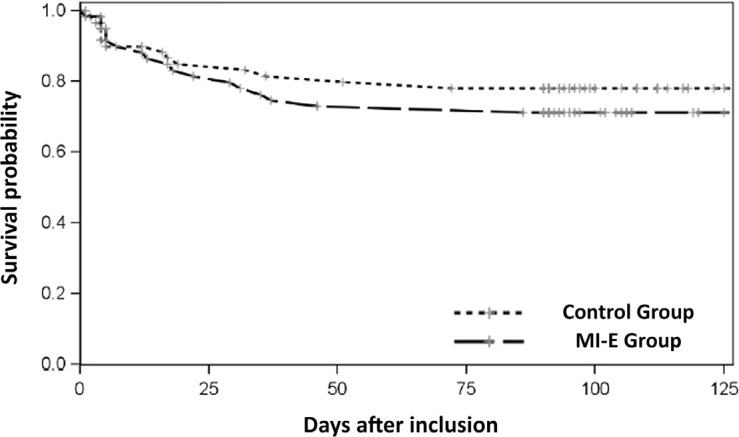
Kaplan-Meier curve representing the survival of patients during follow-up.

The Kaplan‒Meier curve related to mortality until Day 90 (Figure 3) was slightly lower in the MI-E Group, although these results were not significant (p = 0.418). Furthermore, the secondary analysis considering patients with SAPS II scores available at baseline (n = 105) showed that the risk of mortality between the groups was largely reduced after adjustment (HR = 1.61 without adjustment *versus* HR = 1.19 after adjustment).

MI-E group - conventional + mechanical in-exsufflation group.

## DISCUSSION

This is the first study evaluating the efficacy of mechanical insufflation-exsufflation to prevent ARF in a population of intensive care-acquired weakness ICU patients. The combination of CCPT and MI-E to prevent acute respiratory failure 48 hours after extubation in patients with ICUAW seems to be ineffective. Similarly, the reintubation rate, postextubation ICU length of stay, and mortality at Day 90 were also similar. However, less tracheal suctioning was needed in the MI-E Group.

After extubation, ARF may be related to respiratory muscle weakness and a decrease in airway clearance efficiency.^(30-32)^ Consequently, it has been shown that using MI-E results in an improvement in airway clearance in diseases where this clearance is impaired, such as poliomyelitis,^(13)^ Duchenne’s dystrophy,^(14)^ amyotrophic lateral sclerosis^(15,33)^ and other neuromuscular diseases.^(20,34)^ However, a recent Cochrane review concluded that there was not enough evidence to guide the routine use of MI-E in clinical practice in patients with neuromuscular diseases.^(34)^ In our study, ICUAW patients presented a similar condition of muscular weakness, while only a reduced proportion of them presented a high mucus quantity, either in the control group (8%) or in the MI-E Group (10%).

The in-exsufflation pressure set was +40/-40cmH_2_O based on previous studies.^(35)^ Notably, Gonçalves et al. compared MI-E *versus* standard care to improve ARF after extubation in a mixed population of 75 ICU patients using the same range of pressures.^(21)^ They observed a decrease in the number of reintubated patients when MI-E was implemented (17% *versus* 48%; p < 0.05). and a reduction in postextubation ICU length of stay of 6.7 days when MI-E was applied. Our hypothesis is that the differences in results could be related to patient characteristics, as more than 50% of the patients in both groups presented hypoxemic respiratory failure, and the SAPS II level in the study by Gonçalves et al.^(21)^ seems lower than that in this study (~ 20% for the Control Group and ~ 40% in the MI-E Group). It must also be pointed out that in the same study, patients benefited from NIV only if they presented some specific conditions (i.e., respiratory acidosis or respiratory frequency higher than 35bpm), potentially explaining their positive results. Despite the absence of differences in these outcomes in our study, the results obtained, especially in the MI-E group, seem to better reflect recent studies about NIV and ARF postextubation that estimate reintubation in approximately 10 - 20% of patients,^(36,37)^ Gonçalves et al.^(21)^ observed that there was a 48% reintubation in the standard care group. In contrast, we observed a reintubation rate of 3.6% in the Control Group and 10.7% in the MI-E Group, and similar for postextubation ICU length (median of 4 days *versus* mean of 3.1 days, respectively). Moreover, our reintubation rate agrees with previous studies presented in the literature.^(37,38)^

Another aspect to be considered is the implementation of NIV as standard care postextubation in the ICU. Many authors have confirmed that prophylactic and early use of NIV can decrease the incidence of respiratory failure after extubation.^(36,39,40)^ We can hypothesize that in our population of patients, the potential beneficial effect of MI-E may have been diminished or counterbalanced since 78.9% of our cohort was under NIV. However, no data were collected between patients under prophylactic and curative NIV. Further studies assessing both interventions in this population of patients may be interesting.

Acute respiratory failure, reintubation rate and postextubation ICU length are the most correlated outcomes with mortality.^(41,42)^ According to the meta-analysis published by Morrow et al. in 2013,^(34)^ no data are available about mortality in patients with neuromuscular disease using MI-E. In 2015, Mahede et al.^(19)^ reported on an Australian survey a reduction in the risk of death for patients with neuromuscular disease using MI-E at home (median period of 2.5 years). However, it is difficult to make any comparison since the rare studies assessing the effect of MI-E in the ICU were performed during the intubation period and did not analyze mortality during the ICU stay or after discharge.^(43-47)^

Tracheal suctioning is related to major complications such as tachycardia, derecruitment, and tracheal mucosa injury.^(48)^ Bach et al. showed in a study with 46 neuromuscular patients that the amount of tracheal suctioning decreased when MI-E was used. Similarly, our data also showed a significant decrease in the need for at least one suction between interventions (31% in the Control Group, 11.7% in the MI-E Group), which seems to confirm the suggested results by Bach et al.^(13)^ Moreover, these results can impact patient morbidity since suctioning is generally uncomfortable and poorly tolerated.^(49)^

Winck et al. reported a study in 2004 where PCEF was assessed prior to and after MI-E application. In this study, patients with a neuromuscular disorder increased their PCEF from 180 to 220L/minute (p < 0.005).^(33)^ Similarly, two studies led by Bach showed a major increase in PCEF in two different populations of neuromuscular patients.^(13,16)^

However, our results were contradictory to those reported previously. Of note, all our patients were intubated for a short period of time, so we can expect a lower impact of PCEF. Consequently, this could explain the absence of no significance observed in the PCEF evolution in the MI-E Group. Furthermore, PCEF only obtained correct measurements adequate to be analyzed in 66% of the patients, but this parameter was a criterion for exit from the study, since PCEF ≥ 270L/minute was considered a cutoff for an effective cough.^(7,8)^ Consequently, we can expect that some patients were not correctly classified and analyzed.

Several limitations of this study merit consideration. The major limitation of this study was that the sample size was not achieved, thus decreasing the study power as we had a substantially underpower of 12% than the assumed in the sample size calculation, so our non-significant results should be interpreted with precaution.

Despite this, the probability of finding a significant difference in the primary outcome as mentioned above is very weak. Therefore, it seems that the possibility of incurring a type II error is extremely low. A large number of patients using prophylactic NIV after extubation may explain the low ARF rate in the two groups.

The etiologies of acute respiratory failure etiology are heterogeneous and vast. As shown recently by Jaber S et al.^(9)^ the main risk factors for extubation failure due to airway failure are intubation for coma (OR 4.979 (2.797 - 8.864), p < 0.0001), intubation for acute respiratory failure (OR 3.395 (1.877 - 6.138), p < 0.0001), absence of strong cough (OR 1.876 (1.047 - 3.362), p = 0.03), female sex (OR 2.024 (1.187 - 3.450), p = 0.01), length of ventilation > 8 days (OR 1.956 (1.087 - 3.518), p = 0.025) and copious secretions (OR 4.066 (2.268 - 7.292), p < 0.0001). Our cohort does not correspond totally to some of these risk factors; therefore, our assumption that lower ARF rates may mainly be due to secretions is erroneous, as an airway clearance impairment cannot justify its appearance exclusively.

## CONCLUSION

Mechanical insufflation-exsufflation seems to not have any positive effect on preventing postextubation acute respiratory failure, reintubation rate, intensive care unit length of stay and 90-day mortality in patients presenting and intensive care unit-acquired weakness. Given the limitations of our study, potential future clinical studies should carefully reevaluate the targeted population.
